# Hospitals and the Poets.— The Poet Laureate, Henley, and Two Famous Hospitals

**Published:** 1916-01-08

**Authors:** 


### HOSPITALS AND THE POETS.

### The Poet Laureate, Henley, and two Famous Hospitals.

It is unfortunate that the hospital, a centre, like the school, of many hopes, disappointments, and ambitions, unlike the school, has not produced a great book about itself. To see the interplay of personalities which compose its intense and narrow world in the pages of literature we have to turn not to a great novel, like "Tom Brown's Schooldays," but to scattered references here and there among the poets and men of letters who have illuminated, in the course of their writings, now this aspect and now that of hospital life. Consequently, though it is interesting to form an estimate of the extent to which writers, and poets in particular, have turned for their material to the hospital world, such an estimate must be arbitrary, composed as it will necessarily be of numerous scattered allusions, some of which are likely to escape the compiler's glance. Nevertheless, the incompleteness of a review of the relations between the poets and the hospitals is rather a stimulus to our imagination at a time when the Poet Laureate himself is not only a Fellow of the Royal College of Physicians, but was engaged in practice for nearly twenty years, and has held numerous hospital appointments in the course of his professional career.

### A Medical Poet.

The hospitals, then, can claim a Poet Laureate among the famous men of letters whom they have influenced or helped to produce, but, as in practically every instance, it must be added that these years of hospital life have left little mark on the literary work of the writer. Turn, for instance, to our present Laureate's, Mr. Robert Bridges', most popular volume, the *Shorter Poems*, and you will not find there, among the subtle and learned lyrics which fill its pages, any poem, save perhaps one, composed on the rich field of experience which a hospital training might provide to an imaginative observer. Nevertheless, if we cannot find any poetical acknowledgment of Mr. Bridges' debt to hospitals—beyond the interesting fact that he once admitted having always regarded poetry as a kind of learned science, or magic, which could be mastered by a sufficiently careful student—we can at least record what was his personal relation to them.

After being educated at Eton and Oxford, where he graduated M.A. and M.B., Mr. Bridges, after an interval of travel, entered as a medical student at St. Bartholomew's Hospital. Here he became casualty physician. He also held the post of assistant physician at the Children's Hospital, Great Ormond Street. In this connection it is not fanciful to remark on his natural fondness for children—evident enough in his *First Spring Morning* or in that sensitive poem, *On a Dead Child*, where the following verse has, surely, a tender professional touch:

> To me, as I move thee now in the last duty,

> Dost thou with a turn or gesture anon respond;

> Startling my fancy fond

> With a chance attitude of the head, a freak of beauty.

How calm and dignified, but yet tender, is the sentiment implicit in these lines! Another of Mr. Bridges' appointments, by the way, was that of physician at the Great Northern Hospital. Born in 1844 he gained his F.R.C.P. in 1900, although he had retired from practice as far back as 1882. His scientific training, as already hinted, has left its mark on Mr. Bridges' work rather in the spirit in which he has studied the principles of accentuation in English verse (regarding poetry as a kind of divine language to be learnt like the language of the ancient classics), than in the choice or treatment of subject-matter. Lyrics, and lyrical dramas, an oratorio, for he is a learned musician, these with "an account of Milton's prosody" form the body of his work. The latest Laureate is a medical man, but it is *Ulysses* and not Hippocrates who has inspired his song. Nevertheless, his connection with the hospitals has been more intimate and lasting than that of any other poet of the same rank.

In saying above that the hospital, unlike the school, has produced no great book about itself, some enthusiasts may fancy a slight has been placed on William Ernest Henley's well-known work. But though the poems entitled *Rhymes and Rhythms in Hospital* are a most vivid series of impressionist sketches of life in the wards, as viewed from the bed of an observant patient, besides being an example of successful metrical experimentation, they cannot be called a book or a great work. Everybody, one hopes, knows them by this time, but the story of how they came to be composed is too rare an event in the literature of hospital life to be passed over without brief quotation from the verses.

In 1874 Henley, who was in his twenty-sixth year, sought treatment in the Old Infirmary, Edinburgh, for a physical deformity from which he was a sufferer. He was in hospital for some weeks, during which period he composed the well-known *Rhymes and Rhythms*. In these twenty-eight pieces—as in a series of sharp, incisive etchings, bitten in with acid rather than with printers' ink—he describes the events of which each patient is the hero as he enters the hospital, waits, is prepared for the operation, goes to the theatre, awakes from the anæsthetic, forms the subject of a clinical lecture, and is at last discharged. Each event is made the subject of a poem, and the series includes some capital portraits. Could anyone improve on the following description of a patient's sensations under an anæsthetic?

> And you gasp and reel and shudder
> In a rushing, swaying rapture,
> While the voices at your elbow
> Fade—receding—fainter—farther.
>
> Then the lights grow fast and furious,
> And you hear a noise of waters,
> And you wrestle, blind and dizzy,
> In an agony of effort.

Take, again, the few following vivid lines from the poem called *Clinical* in the same series:

. . . the Chief
(His dressers and clerks at attention)
Bends in inspection already.
So shows the ring
Seen from behind round a conjuror
Doing his pitch in the street.
High shoulders, low shoulders, broad shoulders,
narrow ones.
Round, square, and angular, serry and shove;
While from within a voice,
Gravely and weightedly fluent,
Sounds; and then ceases; and suddenly
(Look at the stress of the shoulders!)
Out of a quiver of silence,
Over the hiss of the spray,
Comes a low cry, and the sound
Of breath quick intaken through teeth
Clenched in resolve. And the master
Breaks from the crowd, and goes,
Wiping his hands,
To the next bed, with his pupils
Flocking and whispering behind him.

A capital sketch, as realistic and vivid and unpleasant as such clinical examinations often are to the patient. The patient's feelings have found an authentic voice in this poem. What a chapter in medical history, too, what a memory of Lister's famous and discarded experiment, is contained in the phrase "over the hiss of the spray"! But Henley has eyes for other things besides

Wet, white lint
Brilliantly hideous with red.

He can be tender as well as observant, and has a keen eye for portraiture, as is shown in his wonderful likeness called *The Chief*.

His brow spreads large and placid, and his eye
Is deep and bright, with steady looks that still.
Soft lines of tranquil thought his face fulfil—
His face at once benign and proud and shy.
If envy scout, if ignorance deny,
His faultless patience, his unyielding will,
Beautiful gentleness, and splendid skill,
Innumerable gratitudes reply.

How good that is, and how penetratingly it calls up the face of the great Edinburgh surgeon! Hardly less good is his sketch of the *Scrubber* and of certain patients, but it is unnecessary to quote more. Indeed, the passages above cited are mentioned chiefly to call up delightful literary memories. The patient's point of view, expressed in verse, Henley achieved once and for all. There are many other aspects of hospital life to be observed even by patients, but the broad lines of an average patient's stay in the wards he fixed in poetry with an incisiveness which will not be easily forgotten. It only remains to add that Henley sent these verses to the *Cornhill*, which was edited at that time by Leslie Stephen. Circumstances providing an opportunity, the editor paid a visit to his contributor, which proved to be a great event in Henley's life, since Leslie Stephen brought R. L. Stevenson with him, whose friendship with Henley dated from their meeting in the Old Infirmary ward. All this occurred in 1874. Fourteen years later Mr. H. B. Donkin included Henley's poems in *Voluntaries*, a volume which was itself brought out for an East-End hospital in London. *Rhymes and Rhythms*, by the way, are dated "The Old Infirmary, Edinburgh, 1873-75."

